# Molecular Genetic Variability of Commercial and Wild Accessions of Passion Fruit (*Passiflora* spp.) Targeting *ex Situ* Conservation and Breeding

**DOI:** 10.3390/ijms151222933

**Published:** 2014-12-10

**Authors:** Carlos Bernard M. Cerqueira-Silva, Elisa S. L. Santos, Onildo N. Jesus, João G. P. Vieira, Gustavo M. Mori, Ronan X. Corrêa, Anete P. Souza

**Affiliations:** 1 Laboratory of Applied Molecular Genetics, Department of Exact and Natural Sciences, State University of Southwest Bahia, Itapetinga 45700-000, Brazil; E-Mails: csilva@uesb.edu.br (C.B.M.C.-S.); elisasantosuesb@gmail.com (E.S.L.S.); 2 Molecular Biology and Genetic Engineering Center, University of Campinas, CP 6010 Campinas, Campinas 13083-875, Brazil; E-Mails: jgpvieira@gmail.com (J.G.P.V.); gumori@gmail.com (G.M.M.); 3 Brazilian Agricultural Research Corporation, Cassava & Fruits, Cruz das Almas 44380-000, Brazil; E-Mail: onildo.nunes@embrapa.br; 4 Biotechnology and Genetic Center, Biological Sciences Department, State University of Santa Cruz, Ilhéus 45662-900, Brazil; E-Mail: ronanxc@uesc.br; 5 Plant Biology Department, Biology Institute, University of Campinas, Campinas 13083-875, Brazil

**Keywords:** germplasm, genetic diversity, *Passifloraceae*, molecular markers, single sequence repeats

## Abstract

*Passiflora* species are distributed throughout Latin America, and Brazil and Colombia serve as the centers of diversity for this genus. We performed cross-species amplification to evaluate 109 microsatellite loci in 14 *Passiflora* species and estimated the diversity and genetic structure of *Passiflora cincinnata*, *Passiflora setaceae* and *Passiflora edulis*. A total of 127 accessions, including 85 accessions of *P. edulis*, a commercial species, and 42 accessions of 13 wild species, were examined. The cross-species amplification was effective for obtaining microsatellite loci (average cross-amplification of 70%). The average number of alleles per locus (five) was relatively low, and the average diversity ranged from 0.52 in *P. cincinnata* to 0.32 in *P. setacea*. The Bayesian analyses indicated that the *P. cincinnata* and *P. setacea* accessions were distributed into two groups, and the *P. edulis* accessions were distributed into five groups. Private alleles were identified, and suggestions for core collections are presented. Further collections are necessary, and the information generated may be useful for breeding and conservation.

## 1. Introduction

The terms “passion fruit” and “passion flower” are generally associated with species of the *Passifloraceae* family, particularly those belonging to the genus *Passiflora*. Although the taxonomy is unclear, and thus the total number of species is unknown, the genus *Passiflora* is recognized as the richest genus in terms of the number of species within the family, with approximately 520 species [1]. The vast majority of *Passiflora* species (approximately 96%) are distributed throughout the Americas [2], and Brazil and Colombia are prominent centers of diversity for this genus, as approximately 30% of *Passiflora* species (~150 species in Brazil and ~170 species in Colombia) are found in these regions, with 89 species being endemic to Brazil [3,4,5,6].

Taxonomic and phylogenetic studies, aided by molecular biology techniques, have shown that the genus *Passiflora* can be organized into four subgenera: *Astrophaea*, *Decaloba*, *Deidamiodes* and *Passiflora* [1,7,8,9]. These studies contradict the previous hypotheses that *Passiflora* can be organized into 22 [10] and 23 [11] subgenera. A recent study based on the analyses of genomic sequences as well as plastidial and mitochondrial regions indicates that, historically, *Passiflora* ancestors arrived in Central America and diversified quickly from there, with numerous dispersion events [8]. These authors also argue that the *Passiflora* subgenera diverged approximately ~32 to ~38 million years ago (Mya), which indicates that this diversification is quite ancient. Cytogenetic data on *Passiflora* spp. are still modest, and studies are restricted to less than 20% of species in the genus *Passiflora* [12]. Most *Passiflora* species are diploid, with 2*n* = 12, 18 or 20 [13,14]. Four basic chromosome numbers are considered for passion fruit species, and these are according to the division of the genus into four subgenera: *x* = 6 for *Decaloba*; *x* = 9 for *Passiflora*, with some instances of *x* = 10 or 11; *x* = 10 for *Astrophaea*; and *x* = 12 for *Deidamiodes* [1,13].

Passion fruit species are used as a natural resource for the pharmaceutical [15,16,17], cosmetic [18,19] and juice industries [20,21]; they are also used as ornamental plants [22,23] and consumed as a fresh food item [20,21]. Brazil is the largest producer and consumer of passion fruit [20,24], and production was estimated at 676,000 tons annually for the last 10 years, with approximately 776,000 tons in 2012 alone, totaling approximately 70% of the global supply [20,24,25]. Currently, the average Brazilian productivity is estimated at 14 t·ha^−1^·year^−1^·[25], with the potential to increase up to 50 t·ha^−1^·year^−1^·[26,27]. Therefore, efforts to increase passion fruit crop production, such as the popularization and implementation of appropriate cultivation techniques and management strategies coupled with breeding, are clearly needed.

The passion fruit plant-breeding program established by the Empresa Brasileira de Pesquisa Agropecuária (Embrapa) in Bahia, Brazil has fostered the development of more productive hybrids with good physicochemical fruit characteristics; however, none of the reported varieties exhibit resistance to major pests and diseases. Wild species are potential sources of biotic and abiotic stress resistance genes [28,29,30], and they exhibit extensive phenotypic variation in morpho-agronomic traits [31,32,33,34,35]. Molecular markers are important tools for germplasm characterization because they facilitate different steps in pre-breeding and breeding programs, and they reduce the time required for the release of cultivars. No studies concerning heritable variation have been conducted for most *Passiflora* species, and molecular genetic analysis has been restricted to approximately 15% of the species within the genus, with the majority of studies using only a few accessions per species along with dominant markers [36].

Considering that genetic characterization provides useful information for understanding, conserving and harnessing genetic diversity, thereby allowing for greater interaction between pre-breeding and breeding programs [37,38], the aim of the present study was to measure and describe the genetic variation within *Passiflora*. We evaluated the cross-species amplification of 109 microsatellite markers, also known as single sequence repeats (SSR), in 14 wild and commercial passion fruit species and estimated the intraspecific genetic variability of 116 accessions (364 plants) belonging to three species of *Passiflora* that are recognized to be of interest to plant breeding programs. We then provide suggestions for *Passiflora* breeding and* ex situ* conservation efforts. Finally, we identified the preferred crosses among accessions and the minimum acceptable size for the core collection of each species.

## 2. Results

### 2.1. Tests for Cross-Species Amplification

**Figure 1:**
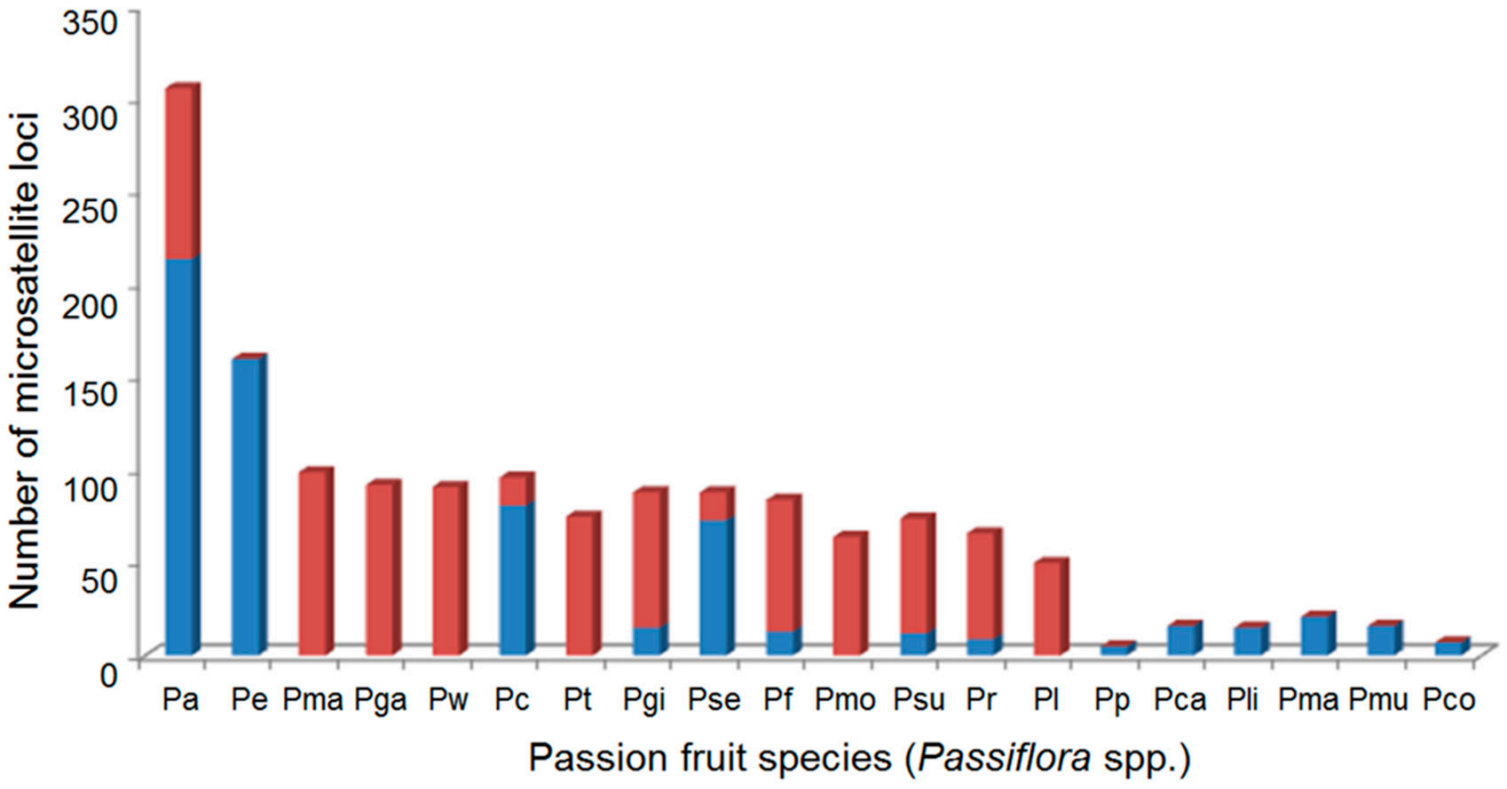
Microsatellite markers available for *Passiflora* species, including markers characterized in this study (red columns) and in previously published studies (blue columns). *Passiflora alata* (Pa), *P. edulis* (Pe), *P. malacophylla* (Pma), *P. galbana* (Pga), *P.* *watsoniana* (Pw), *P. cincinnata* (Pc), *P. tenuifila* (Pt), *P. gibertii* (Pgi), *P. setacea* (Pse), *P. foetida* (Pf), *P. morifolia* (Pmo), *P. suberosa* (Psu), *P. rubra* (Pr), *P. laurifolia* (Pl), *P. pohlii* (Pop), *P. caerulea* (Pca), *P. ligularis* (Pli), *P. maliformis* (Pma), *P. mucronata* (Pmu) and *P. contracta* (Pco).

Cross-species amplification was shown to be an efficient strategy for obtaining microsatellite loci in passion fruit species, with an average cross-amplification success rate of approximately 70% for the 14 *Passiflora* species examined in this study (Table 1). The highest percentages of cross-species amplification (>80%, corresponding to more than 90 SSR loci) were found for *Passiflora alata*, *Passiflora galbana*, *Passiflora malacophylla* and *Passiflora watsoniana*. By contrast, the lowest percentages of cross-species amplification (<60%, corresponding to fewer than 65 SSR loci) were observed for *Passiflora laurifolia*, *Passiflora morifolia*, *Passiflora rubra*, *Passiflora setacea* and *Passiflora suberosa*. We identified a large number of SSR markers for at least 12 new species of passion fruit, with an average of 75 new loci characterized for each species (Figure 1).

We observed a slightly higher average percentage of successful amplifications at loci derived from *P. edulis* and *P. setacea* (≥70%) compared with the loci derived from *P. cincinnata* (<60%) in the different species in this study (Table 1). Moreover, the cross-species amplification of this microsatellite set revealed wide variation. For example, species-specific amplification was observed for three markers (Table S1). Conversely, 23 loci could be amplified from every species tested in this study (Table S1).

**Table 1:** Cross-species amplification observed using a set of 109 primer pairs ^a^ developed for *Passiflora cincinnata* (25 loci), *Passiflora edulis* (32) and *Passiflora setacea* (52) in 14 passion fruit species from the germplasm bank of Embrapa Cassava & Fruits.

*Passiflora* Species	*P. cincinnata*		*P. edulis*		*P. setacea*		Total	
	Loci (*N*)	%	Loci (*N*)	%	Loci (*N*)	%	Loci (*N*)	%
*P. alata* Curtis	19	76	29	91	44	85	92	84
*P. cincinnata* Mast	–	–	24	75	42	81	66	76
*P. edulis* Sims	12	48	–	–	46	88	58	75
*P. foetida* L.	14	56	22	69	35	67	71	65
*P. galbana* Mast	19	76	28	88	45	87	92	84
*P. gibertii* N.E.Br	16	64	21	66	36	69	73	67
*P. laurifolia* L.	12	48	12	38	26	50	50	46
*P. malacophylla* Mast	22	88	30	94	47	90	99	91
*P. morifolia* Mast	12	48	16	50	36	69	64	59
*P. rubra* L.	13	52	16	50	28	54	57	52
*P. setacea* DC.	6	24	21	66	–	–	27	47
*P. suberosa* L.	13	52	19	59	30	58	62	57
*P. tenuifila* Killip	15	60	22	69	38	73	75	69
*P. watsoniana* Mast	18	72	30	94	43	83	91	83
Total	15	59	22	70	38	73	70	68

^a^ Microsatellite markers previously developed by Oliveira [39] and Cerqueira-Silva* et al.* [40,41].

### 2.2. Polymorphism Analysis of Microsatellite Loci

The results of the descriptive statistical analyses of all the SSR loci used for the genetic characterization of *P. cincinnata*, *P. edulis* and *P. setacea* are summarized in Table 2 (for further details, see Tables S2–S4). We identified a total of 355 alleles for the three species evaluated, with 137 alleles for *P. cincinnata* (average of six alleles for the 22 SSR loci examined), 141 alleles for *P. edulis* (average of six alleles for the 23 SSR loci examined) and 77 alleles for *P. setacea* (average of three alleles for the 22 SSR loci examined) (Table 2).

The observed heterozygosity (*H*_O_) values were generally lower than the expected heterozygosity (*H*_E_) values for most (70%) of the SSR loci in the three species of passion fruit. The average values of *H*_O_ and *H*_E_ were 0.42 (ranging from 0.01 to 0.92) and 0.52 (ranging from 0.01 to 0.90), respectively, for *P. cincinnata*; 0.43 (ranging from 0.01 to 0.77) and 0.50 (ranging from 0.01 to 0.86), respectively, for *P. edulis*; and 0.26 (ranging from 0.02 to 0.82) and 0.36 (ranging from 0.02 to 0.69), respectively, for *P. setacea*. Finally, the mean *F* values in the three species were 0.17 for *P. cincinnata*, 0.13 for *P. edulis* and 0.28 for *P. setacea*, and the estimated t values were 0.74, 0.91 and 0.85, respectively.

**Table 2:** Descriptive statistics for the characterization of accessions of *P. cincinnata* (24 accessions and 22 microsatellite loci), *P. edulis* (85 accessions and 23 microsatellite loci) and *P. setacea* (7 accessions and 25 microsatellite loci).

*Passiflora* Species	Values	*N*a	*H*_O_	*H*_E_	*F*	*F*_is_
*P. cincinnata*	Minimum	2	0.00	0.01	−0.41	−0.36
	Maximum	16	0.92	0.89	1.00	0.66
	Average	6	0.42	0.52	0.17	0.15
	Standard deviation	±4.7	±0.2	±0.3	±0.3	±0.07
*P. edulis*	Minimum	2	0.01	0.01	−0.16	−0.27
	Maximum	18	0.77	0.86	0.38	0.31
	Average	6	0.43	0.50	0.13	0.05
	Standard deviation	±3.7	±0.2	±0.3	±0.1	±0.03
*P. setacea*	Minimum	2	0.02	0.02	−0.65	−0.86
	Maximum	6	0.82	0.69	0.87	0.68
	Average	3	0.25	0.36	0.28	0.08
	Standard deviation	±1.4	±0.2	±0.2	±0.4	±0.08

The microsatellite loci were previously identified by Oliveira [39] and Cerqueira-Silva* et al.* [40,41]. *N*a = Number of alleles; *H*_O_ = Observed heterozygosity; *H*_E_ = Expected heterozygosity; *F* = Fixation index; and *F*_is_ = Inbreeding coefficient.

### 2.3. Genetic Structure and Diversity among Passion Fruit Accessions

**Figure 2:**
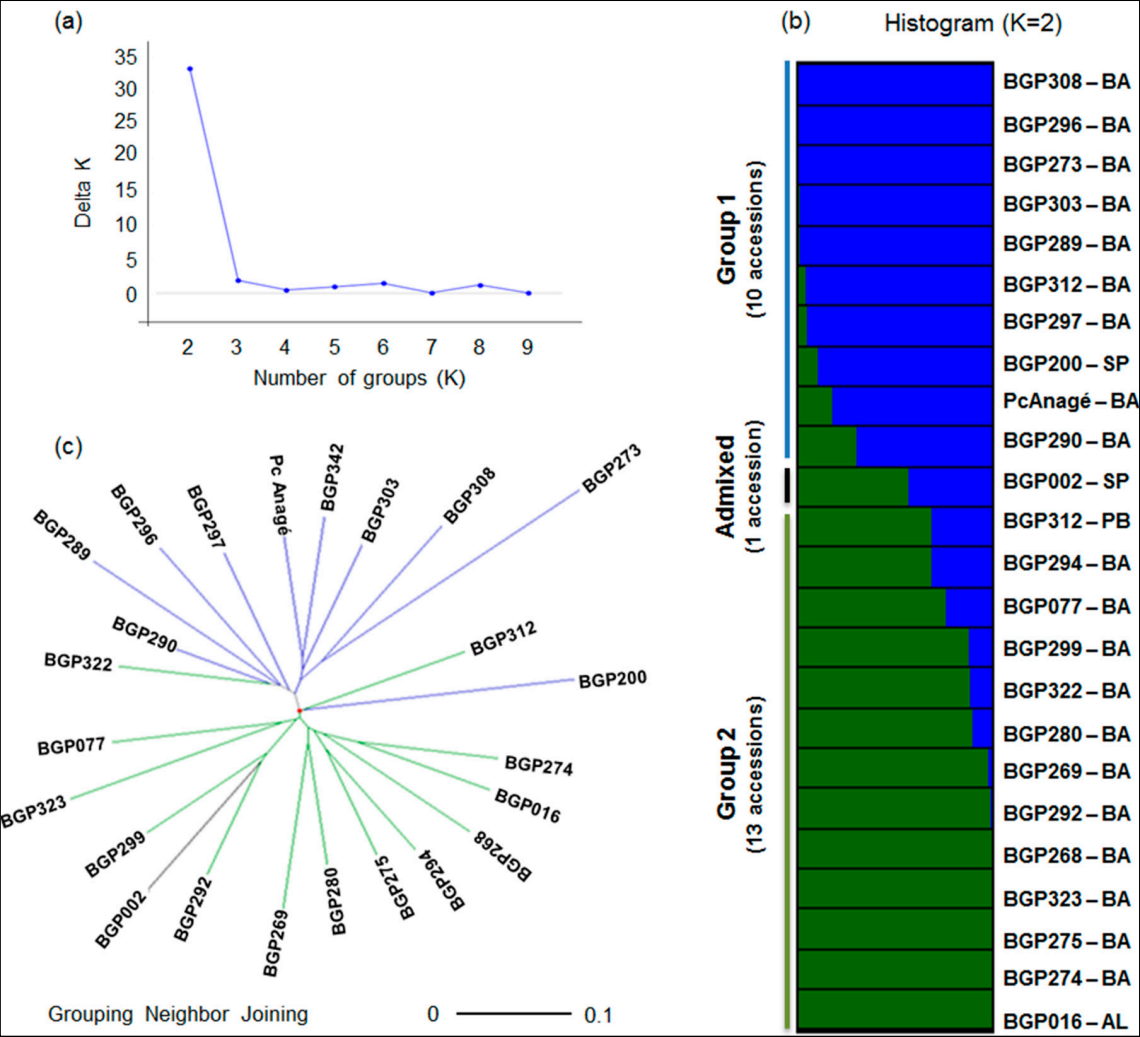
Intraspecific genetic structure of *P. cincinnata*. Clusters were inferred based on Bayesian analyses considering the most probable number of groups (K) estimated with the method described by Evanno* et al.* [43] (**a**); Each column (histogram) (**b**) and line (unrooted neighbor-joining tree) (**c**) represents the genotyping data from three plants. The tree was constructed using Rogers’ genetic distance, as modified by Wright [42], among the 24 passion fruit accessions. The colors used in the histogram and tree represent the most likely ancestry of the cluster from which the accessions were derived.

Bayesian clustering indicated that the 24 accessions of *P. cincinnata* (Figure 2a,b) clustered into two genetic groups (*K* = 2) in which only the BGP002 accession was identified as admixed, with estimated membership (*q*) values of 0.43 and 0.57. The distribution of the accessions in the two suggested groups corresponded to an intermediate level of genetic structure, with a *G*_ST_ value of 0.08. Furthermore, AMOVA indicated that approximately 7% of the observed variation arose from differentiation between these groups (*p* < 0.01).

The average proportion of polymorphic loci (97.5%) did not differ between the groups. However, a large proportion of the identified alleles (41%) were not shared between the two groups, and most of these alleles occurred at a low frequency (below 5%); therefore, these alleles may be considered private and rare alleles (Table 3). We observed contrasting *F* values for groups 1 and 2 (0.20 and 0.09, respectively), which indicated an uneven distribution of alleles in each group and a more substantial reduction of *H*_O_ compared to *H*_E_ in group 2*.* The mean modified Rogers’ distance [42] among the 24 accessions was 0.47, with extreme values recorded for two pairs of accessions: BGP290 and BGP342 (0.28) and BGP273 and BGP323 (0.69). This distance clustering based on the neighbor-joining method supported the distribution of accessions into two groups by the Bayesian method (Figure 2c).

**Figure 3:**
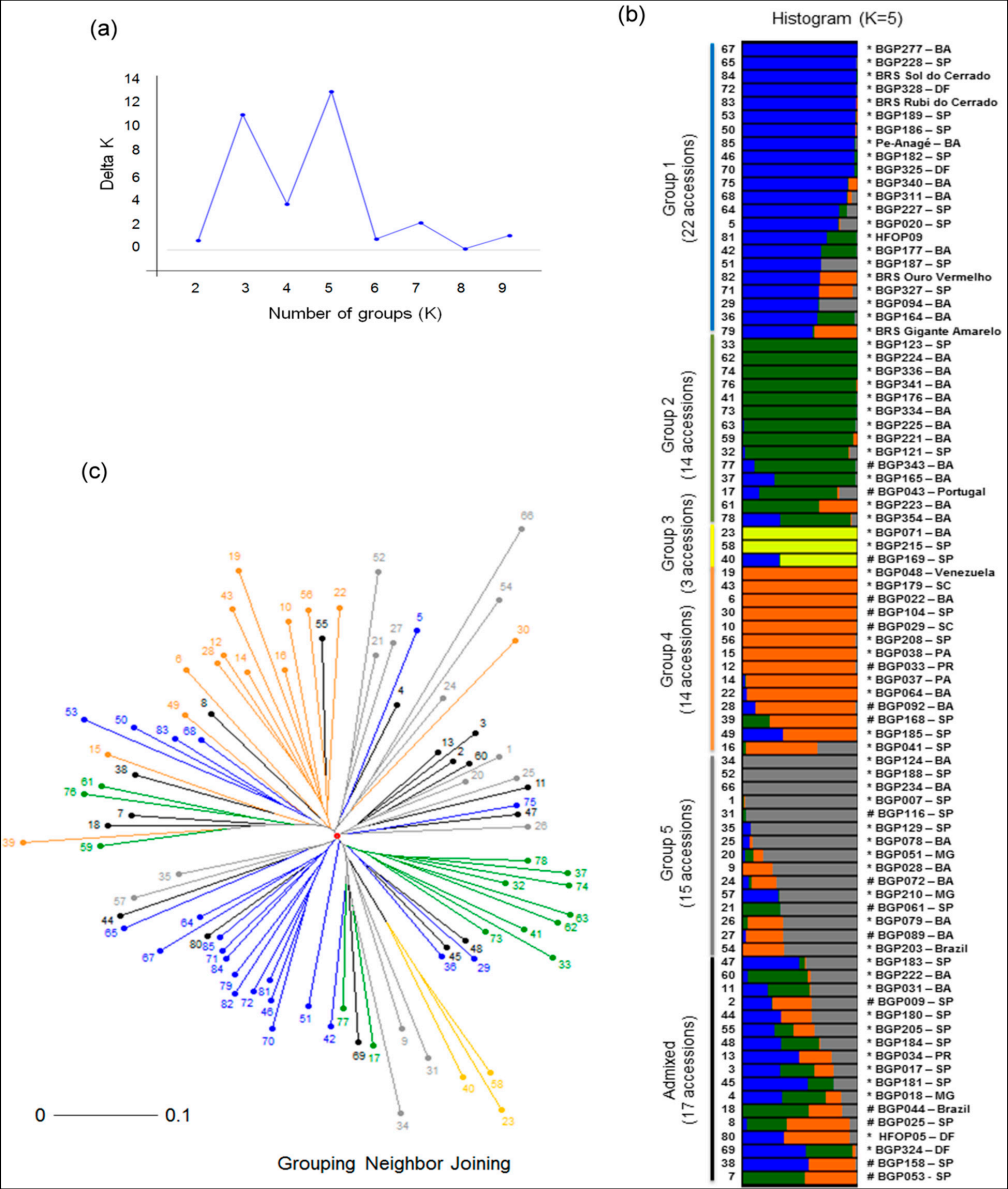
Intraspecific genetic structure of *P. edulis*. Clusters were inferred based on Bayesian analyses considering the most probable number of groups (K) estimated with the method described by Evanno* et al.* [43] (**a**); Each column (histogram) (**b**) and line (unrooted neighbor-joining tree) (**c**) represents the genotyping data from three plants. The tree was constructed using Rogers’ genetic distance, as modified by Wright [42], among 85 passion fruit accessions. The colors used in the histogram and tree represent the most likely ancestry of the cluster from which the accessions were derived. Accessions that produce fruits with yellow and purple peels are indicated with * and #, respectively.

For the 85 *P. edulis* accessions, the Bayesian assignment method revealed five clusters and three clusters as the most likely K’s [43], and these groups were partially concordant with the dendrogram based on the neighbor-joining method (Figure 3c).

The distribution of the accessions among the groups was not homogeneous, and the number of accessions in each group ranged from three to 22. Seventeen accessions were classified as admixed, and none of these accessions had a membership (*q*) value ≥0.6 for a single group. The geographic origins of the accessions did not conclusively determine their membership in a particular group, as indicated by the Bayesian approach (Figure 3b). However, commercial hybrids and cultivars clustered into group 1, indicating that these hybrids and cultivars might have a similar genetic background. The genetic structure estimated based on the formation of the five groups could be considered intermediate (*G*_ST_ = 0.15), and the diversity inherent in the groups corresponded to 10% of the total variance, according to the AMOVA (*p* < 0.01).

The number of polymorphic loci differed among the groups, with percentages varying from 78% (group 3) to 96% (groups 4 and 5). The estimated *F* values ranged from −0.025 (group 1) to 0.145 (group 5). Thirty-one private alleles were identified among the groups estimated using the Bayesian method, and approximately 60% of these alleles were rare alleles with a relative frequency lower than 5% (Table 3). The mean estimated modified Rogers’ distance [42] among the 85 accessions was 0.41, with extreme values recorded for the following pairs of accessions: BGP009 and BGP034, BGP009 and BGP051, BGP007 and BGP222 (with an average distance of 0.21) and BGP071 and BGP234 (with a distance of 0.67).

**Figure 4:**
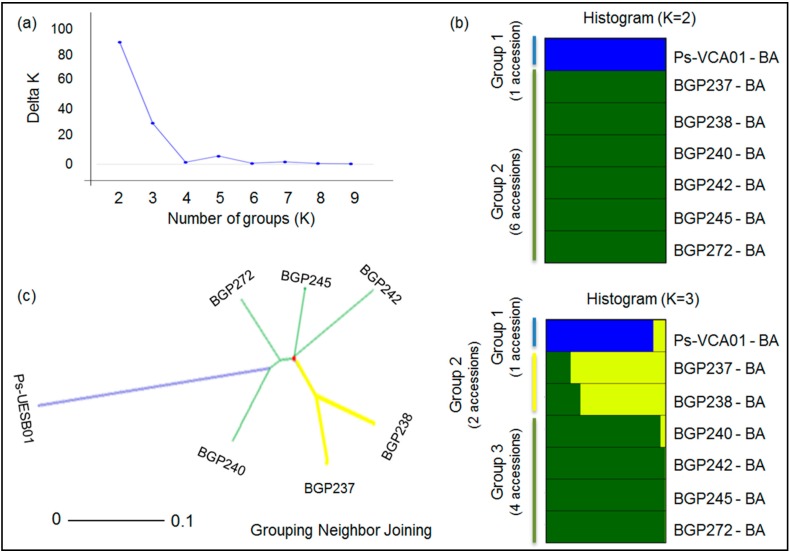
Intraspecific genetic structure of *P. setacea*. Clusters were inferred based on Bayesian analyses considering the most probable number of groups (K) estimated with the method described by Evanno* et al.* [43] (**a**); Each column (histogram) (**b**) and line (unrooted neighbor-joining tree) (**c**) represents the genotyping data from three to 12 plants. The tree was constructed using Rogers’ genetic distance, as modified by Wright [42], among seven passion fruit accessions. The colors used in the histogram and tree represent the most likely ancestry of the cluster from which the accessions were derived.

Considering the seven accessions of *P. setacea*, there are two likely scenarios based on the Bayesian method: *K* = 2 and *K* = 3. Both scenarios were completely compatible with the dendrogram based on the neighbor-joining method (Figure 4). For *K* = 2, all six accessions from Embrapa were grouped together, and only the UESB accession was separated into a distinct group. For *K* = 3, the accessions from Embrapa were divided into two groups, and the UESB accession was retained as a separate group. In the two-cluster scenario, we observed a *G*_ST_ value of 0.36, and the between-group variation corresponded to 36% of the total diversity (*p* < 0.01). However, when *K* = 3, there was a slightly higher *G*_ST_ value (0.38), but a lower proportion of the total variance (29%) was due to differentiation between the inferred groups (*p* < 0.01).

The percentage of polymorphic loci differed between the groups and varied from 92% (group 1) to 64% (group 2) for *K* = 2 and from 92% (group 1) to 52% (group 2) and 60% (group 3) for *K* = 3. The fixation index estimates also differed between the groups and ranged from 0.008 (group 2) to 0.231 (group 1). Thirty alleles may be classified as private and rare alleles, with the vast majority of these alleles restricted to group 1 (Table 3). The estimated mean genetic distance among the seven *P. setacea* accessions was 0.32, with extreme values recorded for the following pairs of accessions: BGP245 and UESB-VCA, BGP272 and UESB-VCA (both with distances of 0.19) and BGP237 and BGP242 (with a distance of 0.54).

**Table 3:** Number of microsatellite loci with private alleles (*N*l), the total number of these alleles (*N*a) and the percentage of rare alleles (*N*a_R_) identified in each genetic group, as estimated based on Bayesian analyses of three passion fruit species.

Groups	*P. cincinnata*			*P. edulis*			*P. setacea*		
	*N*l	*N*a	*N*a_R_	*N*l	*N*a	*N*a_R_	*N*l	*N*a	*N*a_R_
Group 1	10	27	70%	2	2	100%	17	24	25%
Group 2	13	29	69%	1	1	100%	2	2	100%
Group 3	–	–	–	8	13	31%	3	4	50%
Group 4	–	–	–	6	8	75%	–	–	–
Group 5	–	–	–	6	7	75%	–	–	–
Total	16	56	70%	16	31	61%	22	30	27%

### 2.4. Estimates for the Formation of Core Collections

**Figure 5:**
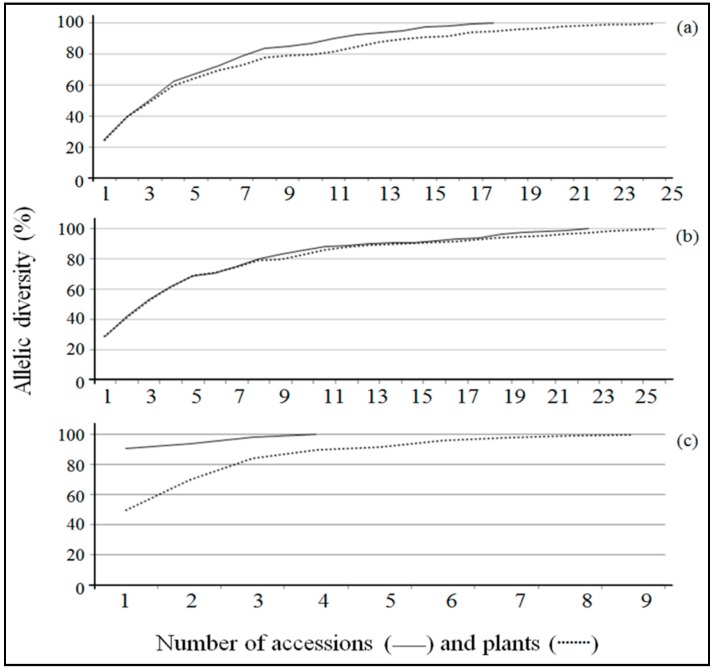
Allelic diversity as a function of the number of plants included in the core collections of three passion fruit species: *P. cincinnata* (**a**), *P. edulis* (**b**) and *P. setacea* (**c**).

The minimum numbers of accessions necessary to represent at least 70% of the allelic diversity present in the germplasm evaluated for *P. cincinnata*, *P. edulis* and *P. setacea* were five, six and one, respectively. Moreover, 18 (25 plants), 23 (26 plants) and 4 (9 plants) accessions were required to ensure that all the alleles identified at SSR loci would be retained in core collections for *P*. *cincinnata*, *P. edulis* and *P. setacea*, respectively (Figure 5 and Table S5).

## 3. Discussion

*Passiflora* pre-breeding programs are of primary importance for the conservation of wild *Passiflora* species, which highlights the importance of characterizing the genetic diversity of *Passiflora* species and the construction and maintenance of active germplasm banks. However, the numbers of accessions/species maintained in collections and germplasm banks are not considered to be fully representative of the genus *Passiflora* [20,44]. In addition, reports have indicated reductions in the numbers of accessions and collections maintained by Brazilian institutions [18], which house many of the largest collections of passion fruit species. Therefore, the availability of representative and well-characterized germplasm banks is critical to the advancement of breeding programs because intraspecific and interspecific hybridization is indispensable for breeding.

In addition to the technical and financial difficulties associated with searching for accessions and maintaining them in germplasm banks, the lack of biological and agronomic information impedes the effective use of the material stored by breeding programs [45]. Therefore, the effective use of germplasm banks at different stages of the breeding process depends on the quality of the work performed during the steps that comprise pre-breeding programs (identifying, characterizing and conserving germplasms) [18,37,46]. In this context, the use of molecular techniques for agronomic characterization tends to maximize the volume and quality of information generated during the pre-breeding stages and reduces the costs and time required for germplasm characterization [45,47].

The assessment of the available genetic variability in germplasm banks is a first step toward the rational conservation and efficient use of genetic resources for plant breeding. This is the first study to investigate the molecular genetic diversity and genetic structure of a large number of commercial and wild accessions of passion fruit, including different species of the genus *Passiflora*. In addition, estimates of the genetic diversity of passion fruit using microsatellites have been obtained only recently and are restricted to studies that monitor diversity in full-sib progenies associated with recurrent selection programs [48,49] or studies of variability using the cross-species amplification of microsatellite loci in a few accessions of passion fruit [50,51].

In summary, our results not only facilitate the use of microsatellite markers for genetic studies of the genus *Passiflora* but also elucidate the genetic structure and diversity of accessions stored in one of the most comprehensive passion fruit germplasm banks (maintained by Embrapa at Cruz das Almas, Bahia, Brazil). Our findings will facilitate discussions of strategies related to gathering expeditions and directed crosses with the aim of developing more efficient breeding programs.

### 3.1. Tests for Cross-Species Amplification

Considering that assessments of microsatellite loci are based on primer pair hybridization to specific regions of the genome (generally tandem repeat sequences of one to six nucleotides), primer pairs designed for source species (*i.e.*, species-specific primer pairs) are typically successfully employed to amplify microsatellite loci in closely related taxa when the DNA regions that flank the microsatellite are sufficiently conserved [41,50,51,52]. Because assessments of microsatellite loci are based on primer pair hybridization to specific regions of the genome, different species are expected to contain some of these conserved regions, albeit in different proportions. The success rate of cross-amplification among the 14 *Passiflora* species evaluated (Table 1) can be considered high compared with the results presented by Paiva [51] (an average of 45%, ranging from 12.5% to 64%) and is more readily comparable to the results reported by Oliveira* et al.* [50] (an average of 67%, ranging from 43% to 100%). The success rates for the cross-species amplification of SSR loci are highly variable among plant species; thus, it is not surprising that variation was observed among the *Passiflora* species evaluated herein [52].

The first cross-species amplification assays in *Passiflora* species were performed by Pádua [9] and Cerqueira-Silva [53]. Together, these authors presented information on the cross-amplification of approximately 100 species. Although considerable in scope, the data reported by Pádua [9] and Cerqueira-Silva [53] are limited in the sense that they provide information on a limited number of SSR loci (approximately 10) for each species evaluated. In addition, these tests were restricted to agarose gel electrophoresis, which limits the evaluation of the amplification profiles of the loci. Recently, more robust studies of cross-amplification were conducted by Oliveira* et al.* [50] using 11 passion fruit species and 21 SSR loci; by Paiva [51] using 10 species and 28 SSR loci; and by Cerqueira-Silva* et al.* [41] using three species and 94 SSR loci. Together, the information provided by evaluations of cross-species amplification in the genus *Passiflora* attests to the effectiveness of this strategy as a means of providing genetic markers for different species of this genus at a reduced cost.

Based on the results obtained using cross-species amplification (Figure 1), there are a substantial number of SSR loci (ranging from 50 to 99 SSR loci) available for the 14 passion fruit species analyzed in this study that are potentially useful for different genetic approaches. However, because only approximately 415 characterized SSR markers are available for the genus *Passiflora* and because these markers were developed from the genomic DNA of only six species (*P. alata*, *P. edulis*, *P. cincinnata*, *P. contracta*, *P. pohlii* and *P. setacea*) [40,41,54,55,56,57,58], both cross-species amplification and the development of new markers are required to advance microsatellite-based genetic studies of the more than 500 species of *Passiflora*.

The identification of loci using cross-species amplification not only enhances intraspecific genetic studies but also aids studies of interspecific diversity. Barbará* et al.* [52] considered SSRs to be one of the most popular and useful markers for ecological studies, and the cross-species amplification of positive characteristics is critical to the success of these studies. In this context, the different subsets of SSR loci conserved among the *Passiflora* species (Table S1) could inspire the first studies of *Passiflora* population genetics as well as studies aimed at characterizing the diversity of wild and commercial accessions found in germplasm banks. Notably, we found that a significant number of SSR loci (23) showed cross-amplification among 14 *Passiflora* species and should thus be considered in interspecific characterizations. Moreover, the identification of sets of SSR loci with different amplification profiles among species (for example, the mPs-Unicamp18, mPe-Unicamp11 and mPe-Unicamp17 loci, which were amplified only in the species of origin) (Table S1) is also of great importance. These loci are useful molecular tools for the confirmation of interspecific hybrids in pre-breeding programs and natural hybrids within sympatric zones.

The use of cross-amplification-derived SSR loci has contributed to the pre-breeding of passion fruit, although the effective use of this approach remains limited because it has only recently been developed. Cross-species amplification to confirm ornamental hybrids derived from crosses among wild species of passion fruit has become a regular component of the activities undertaken in the breeding program developed at the Universidade Estadual de Santa Cruz, Bahia, Brazil [59]. Moreover, characterizations of variability among accessions from germplasm banks using cross-amplification of SSR loci were also initiated in recent years. Interspecific diversity studies were conducted by the research group at the Universidade Estadual do Norte Fluminense, Rio de Janeiro, Brazil, where a total of 56 plants representing 10 species were evaluated using 28 SSR loci [51]. Additionally, in searches conducted as part of the Embrapa breeding program, genetic distances for a total of 61 plants representing 11 species were estimated using 21 SSR loci [50]. With the increase in the number of SSR markers available for passion fruit species, which arise from cross-species amplification tests and the identification of new loci, an increase in the number of studies devoted to characterizing and understanding the diversity of the genus *Passiflora* is also expected.

### 3.2. Polymorphism Analysis of Microsatellite Loci

The genetic diversity observed herein in terms of the number of alleles for *P. edulis*, *P. cincinnata* and *P. setacea* is similar to that reported in previous studies [40,41,55], with a low number of alleles per locus. In addition to this low diversity, the percentage of polymorphic SSR loci observed in studies aimed at developing and characterizing SSR markers for passion fruit did not exceed 35% [40,41,54,58,60,61], with an average of less than 22% polymorphic loci. For example, Ortiz* et al.* [62] did not observe polymorphism when evaluating the germplasm of wild and commercial passion fruit distributed throughout the Colombian territory using 17 SSR primers previously characterized by Oliveira* et al.* [57] and Pádua* et al.* [56]. In this context, Cerqueira-Silva* et al.* [40] reported that the low level of polymorphism in SSR loci appeared to be a pattern among species of the genus *Passiflora*. The genus, however, presents variable agronomic traits, indicating that environmental influences are largely responsible for the morphological changes observed in commercial plantings of passion fruit [6].

The finding that the *H*_O_ values were lower than the *H*_E_ values (Table 3) indicates a tendency toward inbreeding in the evaluated germplasm. Similar results were observed by Oliveira* et al.* [57] for *P. edulis* (average *H*_O_ = 0.58; average *H*_E_ = 0.62), by Pádua* et al.* [56] for *P. alata* (average *H*_O_ = 0.26; average *H*_E_ = 0.53), by Cerqueira-Silva* et al.* [40,41] for *P. cincinnata* (average *H*_O_ = 0.39; average *H*_E_ = 0.44), by Cazé* et al.* [55] for *P. contracta* (average *H*_O_ = 0.53; average *H*_E_ = 0.61) and by Cerqueira-Silva* et al.* [41] for *P. setacea* (average *H*_O_ = 0.34; average *H*_E_ = 0.41).

**Figure 6:**
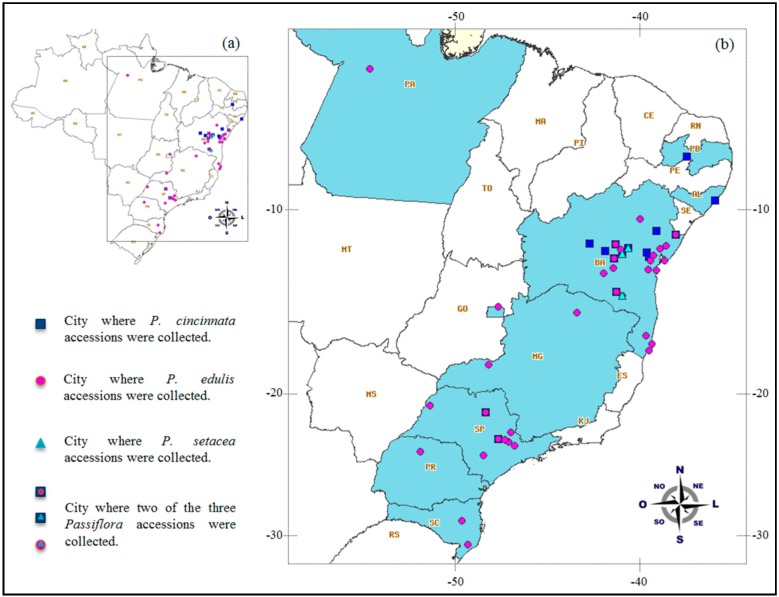
Map of Brazil (**a**) highlighting the geographical areas from which the passion fruit accessions (*Passiflora* spp.) were sampled; and (**b**) location of the accessions plotted on a map using the SpeciesMapper tool, which is available at SpeciesLink [78].

The mean *F* values in all three species (0.17, 0.13 and 0.28 for *P. cincinnata*, *P. edulis* and *P. setacea*, respectively) reinforce the idea of the loss of heterozygosity, as suggested by the values of *H*_O_ and *H*_E_, which is more evident for *P. setacea*. These results may be partially explained by the origins of the accessions present in germplasm banks, as all of the *P. setacea* accessions and approximately 79% and 36% of the *P. cincinnata* and* P. edulis* accessions, respectively, were from Bahia (Figure 6 and Table S6). As expected, because these are cross-pollinated species [63], their estimated outcrossing rate (calculated from the *F*_IS_ values) was high (*t* = 0.84).

### 3.3. Genetic Diversity and Structure among Passion Fruit Accessions

Given that the accessions examined in this study do not represent the entire natural geographic distribution of *P. edulis*, *P. cincinnata* and *P. setacea* and that most of the accessions (approximately 70%) were from only two Brazilian states (Bahia and São Paulo), the genetic groups defined by the STRUCTURE program could not be associated with geographic regions. Thus, for population studies of the genetic diversity of passion fruit, new samples must be acquired from natural populations, particularly for *P. cincinnata* and *P. setacea*, which were less commonly represented in germplasm banks. Studies of diversity among passion fruit accessions based on dominant markers [64,65] failed to find an association between estimated genetic diversity and geographic origin. In these studies, the authors considered the high flow of biological material among producers as a likely factor contributing to a lack of association between the genetic diversity and geographic origin of the accessions. The flow of biological material and the self-incompatibility of passion fruit, which both favor the exchange of alleles, were possible causes of the failure of STRUCTURE to define groups for seventeen *P. edulis* accessions (Figure 3b). These accessions likely have multiple ancestries compared with other accessions.

The formation of two groups for *P. cincinnata* using the Bayesian clustering method was corroborated by the tree constructed using the neighbor-joining method, highlighting the distribution of accessions from the Bahia state and the clustering of the accessions from the Alagoas and Pernambuco states (both in northeastern Brazil) in the same group (Figure 2). The distribution of *P. setacea* accessions in two or three groups, which was suggested by STRUCTURE and corroborated by the neighbor-joining tree, reinforces the hypothesis that a relatively low number of accessions are represented in relation to the diversity of natural species. We highlight the fact that six of the seven accessions (BGP237, 238, 240, 242, 245 and 272, totaling 38 plants) presented low genetic diversity values (68% polymorphic loci) and few private alleles (six, representing only 20% of the private alleles identified) compared with the values observed for the Ps-UESB01 accession (12 plants), which showed 92% polymorphic loci and 24 private alleles (representing 80% of the private alleles identified). The high genetic variability among *P. setacea* accessions from fragments of native forest in the southwest region of Bahia (fragments of the Forest of Liana, 14°53'S and 40°47'W) was also observed by Cerqueira-Silva* et al.* [66].

Taken together, the results reveal that both *P. cincinnata* and *P. setacea* were undersampled in the evaluated germplasm and that diversity has maintained high levels of genetic structure, indicating the potential occurrence of diversity loss due to genetic drift in this material. Future efforts should be directed toward increasing the representation of these two species in collections to aid both plant breeding [26,30] and *ex situ* conservation [37,38,53] efforts. Regarding the conservation of *P. cincinnata* and *P. setacea*, it is noteworthy that studies of accessions obtained from fragments of degraded native forest reported low variability among accessions of *P. cincinnata* [67]. A similar study identified significant variability in *P. setacea* [66]; however, this species is endemic to the Brazilian territory [68].

The distribution of *P. edulis* accessions in the groups estimated using the Bayesian approach and corroborated by the neighbor-joining method led to the significant finding that five of the six hybrids and cultivars evaluated were clustered in the same group (Figure 3b). These data provide evidence of the homogeneity of this material, indicating that the hybrids in this group could have a similar genetic background. In support of this possibility, the parents of the HFOP09 hybrid are the BGP165 accession and the “BRS Gigante Amarelo” variety. In addition, low genetic diversity in commercial accessions of this species was also suggested by different authors [6,46,62]. These results corroborate the hypothesis of a narrowing in the genetic base of the available commercial cultivars and accessions of yellow passion fruit [6,46,69]. The low diversity observed in *P. edulis*, particularly among commercial accessions, could be due to germplasm exchange within Brazil because in most cases, passion fruit producers generate their own seedlings, either from seeds collected in their neighborhood or from fresh fruits purchased at the market [64].

Therefore, we highlight the need to increase the genetic variability in collections composed of commercial accessions via the addition of wild accessions that will improve breeding programs [6,46]. However, it is not sufficient to increase the number of accessions stored in germplasm banks because, as discussed by Freitas* et al.* [70], the dearth of information due to the lack of characterization is one of the major obstacles to the effective use of new accessions in breeding programs. These authors argued that as a consequence of this lack of information, breeders are encouraged to continue exploring small collections, which consequently limits the use of germplasm and leads to a narrowing of the genetic base of cultivated passion fruit. The low genetic diversity in active collections can undermine the advancement of breeding programs and the effective *ex situ* conservation of the diversity in natural populations. In this context, the low genetic variability present in passion fruit cultivars may contribute to the susceptibility of this material to the most important diseases affecting the crop [29]. Considering the potential use of wild accessions as a source of genetic variability and the limited representation of accessions from different geographic regions in the evaluated germplasm relative to the natural distribution of passion fruit species (Table 4 and Figure 6), additional collecting expeditions are required.

Regarding diversity studies conducted in *P. edulis*, Bellon* et al.* [24] observed greater genetic distance between accessions of fruits with yellow and purple peels. In the germplasm evaluated in this study, 18 of 85 accessions had fruits with purple peels and were represented in four of the five groups inferred using the Bayesian analysis (Figure 3b). Although there was no clear distinction between groups of accessions with yellow and purple fruits, we observed 37 private alleles in accessions of yellow passion fruit (approximately 26% of the alleles identified in the germplasm). Similar results were observed in molecular characterizations performed with microsatellite markers among accessions of yellow and purple passion fruit (*P. edulis*) [71]. These authors observed that approximately 30% of the alleles were private, which suggests that these alleles could be useful in monitoring hybrids between yellow and purple passion fruit.

Although these accessions are recognized as the same species [72], these results reinforce the need to distinguish accessions of *P. edulis* by the color of their fruit [24]. This variation may be understood as a phenotypic marker of more robust genetic variability. Microsatellite loci with the most private alleles identified among accessions of fruits with purple and yellow peels (mPe-UNICAMP02, Pe-19, Pe-20, Pe-23, Pe-24, Pe-42, Pe-58, mPc-UNICAMP11, mPs-UNICAMP09 and mPs-UNICAMP16) could be prioritized in future studies to characterize and monitor variability among progeny derived from accessions with contrasting fruit traits. Considering that *P. edulis* has at least 150 characterized SSR loci, it would be possible to build and characterize a mapping population to elucidate the genetic architecture of fruit traits. Thus, marker-assisted breeding, at least for *P. edulis* and *P. alata*, is fairly plausible over the medium- and long-term because of the commercial interest in these species and because of the large number of available SSR markers.

Estimates of genetic distance also contribute to breeders’ decisions regarding the selection and crosses of divergent and convergent parents [47,65]. In this context, molecular markers could aid in the estimation of the difference in alleles present in a population, as well as in germplasm banks, and could enhance the genetic gains derived from crosses that explore the maximum heterosis effect. The use of molecular markers in passion fruit breeding programs has been encouraged [49]. Recent results describing the characterization and selection of yellow and purple passion fruit accessions based on microsatellite markers and disease reactions allowed for the identification of preferred crosses. The progeny obtained from these preferred crosses are likely to have enhanced disease resistance and be more representative of the available genetic variability [71].

In the present study, we identified many intraspecific crosses (convergent and divergent) that increase (BGP071 and BGP234, BGP273 and BGP323, BGP237 and BGP242) or decrease (BGP009 and BGP034, BGP009 and BGP051, BGP007 and BGP222, BGP290 and BGP342, BGP245 and UESB-VCA, BGP272 and UESB-VCA) the available genetic variability in the evaluated accessions. Obviously, information on the agronomic traits of interest should also be considered during the selection of preferred crosses as well as during the selection of segregating progeny. Although such crosses are limited by the lack of agronomic information for most of the genotyped accessions [70], we recommend the use of genotypes that will generate segregating progeny with the potential for use in the fresh fruit trade and in the production of juice.

BGP185 and BGP051, which are promising accessions for the fresh fruit market [70], were placed in different groups by the STRUCTURE program and were shown to be divergent in the grouping performed by the neighbor-joining method (Figure 3b,c). Therefore, in addition to possessing desirable agronomic characteristics, crosses between these accessions may produce progeny with desirable variability for future stages of selection and/or progeny that exhibit the positive effects of heterosis. Moreover, when considering the accessions that are promising for use in the production of juice [70] in combination with the diversity estimated using microsatellite markers (Figure 3b,c), it is possible to identify both divergent (BGP034 and 181 or BGP0123, BGP0079 and BGP123 or 181) and convergent crosses (BGP034 and BGP079, BGP123 and BGP181). We believe that information derived from future agronomic evaluations (regarding both the physicochemical characteristics of fruits and disease resistance) and the molecular genetic diversity data generated in this study will enable a more conscious use of the variability available in germplasm banks.

Finally, considering the lack of direct relationships between geographical and genetic distances and the high levels of inbreeding (relative to the fixation index) observed for the three species considered in this study (Table 2), we propose that expeditions to obtain new accessions must consider the different biomes. We understand that the collection of a larger number of plants should be prioritized at the expense of the number of fruits/seeds per plant. In addition, the actions of different pollinators of *P. setacea* (pollinated by bats) and *P. cincinnata* and *P. edulis* (pollinated by bees) and their dispersal areas should be taken into account when establishing collection strategies. Moreover, additional information regarding the ancestry of commercial materials should be prioritized when adding new accessions to germplasm banks. The characteristics inherent in the process of commercial cultivation of the passion fruit crop in Brazil, including the fact that seeds are procured largely by the producers and the flow of material between crop regions [64], can lead to redundancy in the genetic information available for the accessions that should be maintained in germplasm banks.

### 3.4. Formation of the Core Collection

The COREFINDER analysis showed that for the three passion fruit species, the entire set of alleles found in this study could be represented by core collections with 75%, 27% and 57% of the accessions from *P. cincinnata*, *P. edulis* and *P. setacea*, respectively (Figure 5). In addition, only 10% of the 116 accessions used to represent the three *Passiflora* species were indicated as sufficient to maintain 70% of the alleles identified in this study. The difference observed between the number of accessions and plants required to retain all the alleles identified in core collections (Figure 5 and Table S5) indicates the existence of significant variability among plants of the same accession. Because passion fruit is allogamous, due to its self-incompatibility [63], genetic variability is expected even among plants of the same accession (even if they are the progeny of controlled pollination between related plants). A similar result was previously observed in accessions of commercial species (*P. edulis* and *P. alata*) [64].

Due to the potential gene flow among passion fruit accessions, special attention should be devoted to strategies for managing and restoring the active germplasm banks of passion fruit to avoid contamination among accessions and allelic loss. To achieve this goal, variability among the accessions should be monitored, and allelic loss and effective population size should be studied [64].

The core collection indicated for *P. cincinnata* included accessions from all three Brazilian states, whereas the core collection suggested for *P. edulis* included accessions from four of the seven represented Brazilian states (Bahia, São Paulo, Minas Gerais and Pará) and one accession from Portugal. Accessions from the states of Paraná, Santa Catarina or the Federal District, as well as the accession from Venezuela, were not included in the collection proposed for *P. edulis*. The failure to include accessions from certain geographical regions does not necessarily reflect low genetic variability in these regions or redundancy among accessions because the sampling was highly uneven among geographic regions.

Certain factors may have contributed to the different numbers of accessions in the core collections suggested here, including the different numbers of accessions evaluated for each species and the differences in the geographical origins of representative accessions of each species. We believe that the prevalence of accessions collected from one or a few geographic regions may, to some extent, compromise the genetic diversity present in the germplasm bank, resulting in a reduced number of individuals compared with the number that is necessary to fully represent allelic diversity Santos-Garcia* et al.* [73], who used genetic diversity information to establish core collections of important forage legumes from tropical and subtropical regions, noted that the sample composition of a germplasm bank could lead to a reduced number of accessions that are representative of a core collection.

According to the general criteria required to form a core collection [37,74,75], 5% to 20% of the available germplasm that represents at least 70% of the total diversity should be retained. In this context, the core collection proposed for *P. edulis* is adequate, as 27% of the characterized accessions are maintained (at minimum) and 100% of the allelic diversity can be estimated via the SSR loci. However, the scenario is different for *P. cincinnata* and *P. setacea*. Although the suggested core collection can also adequately represent variability due to the low number of accessions characterized in the germplasm and the limited representation of accessions from different geographic regions in the evaluated germplasm relative to the natural distribution of passion fruit species, it is advisable to increase the number of accessions in core collections of these passion fruit species. This information, coupled with phenotypic data for traits of interest (such as high productivity and resistance to both abiotic factors and disease), would help to more effectively identify the accessions that should be maintained in a core collection.

## 4. Experimental Section

### 4.1. Germplasm Material and Genomic DNA Extraction

A total of 127 passion fruit accessions were analyzed, including three accessions from the Universidade Estadual do Sudoeste da Bahia (UESB) collection, Itapetinga, Bahia, Brazil and 124 accessions from the germplasm collection of Embrapa Cassava & Fruits, Cruz das Almas, Bahia, Brazil, which included 85 accessions of the commercial species *P. edulis* Sims (yellow and purple) and 42 accessions of 13 wild species of *Passiflora*. For each accession, we used an average of three plants (ranging from one to 12 plants per accession). Table 4 and Figure 6 present general information about the germplasm used, such as the number and origins of the accessions and the genotypes of each species. Detailed information regarding each accession is available in the supplementary material (Table S6). The genomic DNA was isolated from fresh leaves using the cetyltrimethylammonium bromide (CTAB) method [76].

### 4.2. Analysis of Molecular Markers

A set of 109 SSR loci was used at different stages in this study. This set included 15, 25 and 69 loci that were originally identified and characterized by Oliveira [39], Cerqueira-Silva* et al.* [40] and Cerqueira-Silva* et al.* [41], respectively. Diversity estimates and a cross-species amplification test of the SSR loci were performed using polymerase chain reaction (PCR) in a final volume of 15 μL containing 12 ng of template DNA, 0.4 mM each primer (reverse and forward) and 1.0 U of Taq DNA polymerase (Invitrogen Co., Carlsbad, CA, USA). The concentrations of the other reagents (dNTPs, MgCl_2_ and buffers) and the amplification conditions (annealing temperature and PCR cycle characteristics) were described previously [39,40,41].

The amplification products were initially separated by 3% (*m*/*v*) agarose gel electrophoresis with 0.5× TBE (Tris-borate-EDTA) buffer and stained with ethidium bromide. To characterize the amplification profiles, we then performed vertical electrophoresis on 6% (*m*/*v*) denaturing polyacrylamide gels with 1× TBE buffer, and the gels were stained with silver nitrate [77]. A 10-bp DNA ladder (Invitrogen) was used as a reference to determine the product sizes. We evaluated the reproducibility of the electrophoretic profiles by re-genotyping a minimum of 10% of the samples.

**Table 4:** General information about the 14 passion fruit species characterized in this study.

*Passiflora* Species	Popular Name in Brazil	Natural Occurrence (Brazilian Macro-Regions)	Total Number of Accessions ^b^	Total Number of Plants	Geographical Origin
*P. cincinnata* Mast	maracujá do mato/mochila	North—Northeast—Midwest—Southeast	24	67	Alagoas, Bahia, São Paulo, Paraíba
*P. edulis* Sims	maracujá azedo/amarelo	North—Northeast—Midwest—Southeast—South	85	247	Bahia, Distrito Federal, Minas Gerais, São Paulo, Santa Catarina, Pará, Paraná, Portugal, Venezuela
*P. setacea* DC. ^a^	maracujá do sono/sururuca	Northeast—Midwest—Southeast	7	50	Bahia
*P. alata* Curtis ^a^	maracujá doce	North—Northeast—Midwest—Southeast—South	1	3	Distrito Federal
*P. gibertii* N.E.Br	–	Midwest	1	3	Unavailable
*P. laurifolia* L.	maracujá laranja/perola	North—Northeast—Midwest	1	3	Unavailable
*P. tenuifila* Killip ^a^	maracujá de cobra	South—Southeast	1	3	São Paulo
*P. morifolia* Mast	maracujá da capoeira/peludo	Midwest—Southeast—South	1	3	São Paulo
*P. galbana* Mast ^a^	maracujá luxo	Northeast—Southeast	1	3	Unavailable
*P. watsoniana* Mast ^a^	–	Northeast—Southeast	1	3	Unavailable
*P. rubra* L.	maracujá de estalo	Northeast—Southeast	1	3	São Paulo
*P. suberosa* L.	maracujá cortiça/maracujazinho	Northeast—Midwest—Southeast—South	1	3	São Paulo
*P. foetida* L.	–	North—Northeast—Midwest—Southeast—South	1	3	São Paulo
*P. malacophylla* Mast ^a^	–	Northeast—Southeast	1	3	Unavailable

^a^ Species endemic to Brazilian territories [68]; ^b^ with the exception of two accessions (one accession each of *P. cincinnata* and *P. setacea*, which are present in the collection of the UESB, Itapetinga, Bahia, Brazil), all 125 accessions are maintained in the germplasm bank of the Embrapa Cassava & Fruits/EMBRAPA, Cruz das Almas, Bahia, Brazil.

### 4.3. Cross-Species Amplification and Characterization of Intraspecific Genetic Diversity

Cross-species amplification of the 109 SSR loci in the 14 *Passiflora* species considered in this study was recorded as a binary value (*i.e.*, the presence (1) or absence (0) of bands). Descriptive analyses associated with the cross-amplifications were performed, and we calculated the percentage of cross-amplifications by species and loci. The mean values and standard deviations were recorded.

We studied the organization of the genetic variation in three species that are of great interest to passion fruit breeding programs: the wild species *P. cincinnata* (67 plants, representing 24 accessions) and *P. setacea* (50 plants, representing seven accessions) and the commercial species *P. edulis* (247 plants, representing 85 accessions) (Table 4 and Table S6; Figure 6). For this purpose, we used different estimates of intra-species genetic diversity and structure. For each species, we performed a descriptive statistical analysis for all the SSR loci, and the number of alleles per locus (*N*a), observed heterozygosity (*H*_O_), expected heterozygosity (*H*_E_) and fixation index (*F*) were calculated using GenAlEx v. 6.5 [79]. The apparent outcrossing rate (*t*) was calculated from the inbreeding coefficient (*F*_IS_) using the equation *t* = (1 − *F*_IS_)/(1 + *F*_IS_) [80]. Moreover, deviations from Hardy-Weinberg equilibrium and the occurrence of linkage disequilibrium were evaluated using GENEPOP software [81]. The significance level was adjusted using the sequential Bonferroni correction for multiple comparisons.

Analyses of population structure were performed with the Bayesian method using the STRUCTURE software, version 2.3.4 [82,83]. Considering that the present study was conducted using accessions (comprising plants from controlled crosses) maintained in a germplasm bank, we used a no-admixture model with independent allele frequencies in each population. The burn-in period and replication numbers were set to 100,000 and 1,000,000, respectively, for each run. The number of groups (*K*) was systematically varied from 1 to 10, and 20 simulations were performed to estimate each *K*. We used the Δ*K* *ad hoc* method described by Evanno* et al.* [43] and implemented in the online tool Structure Harvester [84] to estimate the most likely *K* in each set of passion fruit species. After estimating the most likely *K*, we used the greedy algorithm implemented in CLUMPP v.1.1.1 [85] with a random input order and 1000 permutations to align the runs. The results were visualized using DISTRUCT v.1.1 [86]. Based on the posterior probability of membership (*q*) of a given accession belonging to a given group compared to the total number of groups (*K*), we classified individuals with *q* > 0.60 as a member of a given cluster, whereas for clusters with membership (*q*) values ≤0.60, the accession was classified as admixed.

The modified Rogers’ distance [42] was calculated using Tools for Population Genetic Analysis (TFPGA) software [87], and the result was used to infer the genetic relationships among accessions using the UPGMA criteria. These relationships were also studied by analyzing genotypic data using the neighbor-joining clustering method in DARwin 5.0 [88]. A hierarchical analysis of molecular variance (AMOVA) and Nei’s GST among the groups, as defined by the STRUCTURE analysis, was performed using GenAlEx v.6.5 [79]. This software was also used to identify private alleles (*i.e.*, alleles that occurred in only one of the groups suggested by the Bayesian analysis) and rare alleles (*i.e.*, alleles with a frequency of occurrence lower than 5%) in the estimated clusters for each species.

Finally, we used COREFINDER [89] to assemble core collections estimated to represent both 70% and 100% of the genetic diversity present within each of the three species.

## 5. Conclusions

Considering the high percentage of success observed in the use of cross-species amplification reactions to obtain microsatellite loci in passion fruit species, cross-amplification appears to be an efficient and low-cost strategy for molecular genetic studies in the genus *Passiflora*. The data indicate the need for further collection expeditions, particularly for wild *Passiflora* species (*P. cincinnata* and *P. setacea*), and the diversity estimates confirm the low levels of genetic polymorphism in microsatellite loci characterized in species of the genus *Passiflora*. The data generated by this study may be useful for breeding programs, conservation, and preferential crosses based on genetic estimates and agronomic analyses.

## Supplementary Materials

Supplementary tables can be found at http://www.mdpi.com/1422-0067/15/12/22933/s1.
